# Assessment of vital organ oxygenation by near-infrared spectroscopy in pharmacologic closure therapy for patent ductus arteriosus in preterm neonates

**DOI:** 10.1590/1806-9282.20251868

**Published:** 2026-06-29

**Authors:** Zuleykha Ziyadova, Murat Konak, Alaaddin Yorulmaz, Melih Timuçin Doğan, Ahmet Sert, Saime Sündüs Uygun, Hanifi Soylu

**Affiliations:** 1 Selçuk University, Selçuklu Medical Faculty, Department of Pediatrics – Konya, Turkey.; 2 Selçuk University, Selçuklu Medical Faculty, Department of Pediatrics, Division of Neonatology – Konya, Turkey.; 3 Selçuk University, Selçuklu Medical Faculty, Department of Pediatrics, Division of Pediatric Cardiology – Konya, Turkey.

**Keywords:** Ductus arteriosus, patent, Infant, premature, Spectroscopy, near-infrared, Splanchnic circulation, Cerebrovascular circulation, Renal circulation

## Abstract

**OBJECTIVE:**

Hemodynamically significant patent ductus arteriosus is a frequent problem in preterm neonates and may impair systemic and organ-specific oxygenation. Near-infrared spectroscopy provides a non-invasive tool to evaluate regional tissue oxygenation. The aim of this study was to investigate the effects of pharmacological closure therapy on cerebral, renal, and mesenteric tissue oxygenation in preterm neonates diagnosed with hemodynamically significant patent ductus arteriosus.

**METHODS:**

This prospective observational study was conducted in a tertiary neonatal intensive care unit between September 2021 and November 2024. Twenty-four preterm infants (24–34 weeks’ gestational age) with hemodynamically significant patent ductus arteriosus confirmed by transthoracic echocardiography were enrolled. Paracetamol treatment was given in cases where ibuprofen was contraindicated. Patients who underwent medical patent ductus arteriosus closure were divided into two groups: ibuprofen and paracetamol groups. Specific regional tissue oxygen saturation and fractional tissue oxygen extraction in three regions were monitored continuously using near-infrared spectroscopy for 72 h before and after medical treatment. Echocardiographic and hemodynamic parameters were recorded.

**RESULTS:**

While patent ductus arteriosus closure was achieved in 46% of cases with medical treatment, a significant reduction in patent ductus arteriosus diameter was achieved in 29% of cases. Ibuprofen was superior to paracetamol in reducing patent ductus arteriosus diameter. Mesenteric regional tissue oxygen saturation also improved significantly at 24–48 h in the ibuprofen group (p=0.01), while cerebral and renal oxygenation remained unchanged (p>0.05).

**CONCLUSION:**

Our findings revealed that ibuprofen therapy improved hemodynamic stability and mesenteric oxygenation in preterm neonates with hemodynamically significant patent ductus arteriosus. Although we showed in our study that there was no significant change in cerebral and renal oxygenation of hemodynamically significant patent ductus arteriosus with ibuprofen treatment, more comprehensive studies showing the changes with near-infrared spectroscopy are needed.

## INTRODUCTION

A patent ductus arteriosus (PDA) is common in preterm babies and affects their blood supply. It occurs 30–70% of the time. A significant PDA (hsPDA) allows blood to flow the wrong way through the heart, and can cause problems, including impaired blood flow to the brain, kidneys, and gut. This may lead to respiratory failure, bronchopulmonary dysplasia, bleeding in the brain, and death. So, early diagnosis and treatment of hsPDA are important^1–3^. High levels of prostaglandins may delay the closure of the ductus arteriosus (DA) in preterm babies. Ibuprofen, paracetamol, and indomethacin are often used to close the DA by inhibiting prostaglandin synthesis^4,5^.

Echocardiography (ECHO) is the gold standard for diagnosing and assessing hemodynamic significance, but it provides limited insight into regional perfusion^6,7^. Near infrared spectroscopy (NIRS) allows non-invasive, continuous monitoring of regional tissue oxygenation (rSO_2_) and fractional tissue oxygen extraction (FTOE), offering complementary data on end-organ impact^8^. NIRS shows if tissues (e.g., the brain) have enough oxygen and how much oxygen is used. Together with FTOE, it can show how active the organ is and how much oxygen it needs^9^.

Although several prior studies have examined perfusion changes before and after PDA treatment, findings remain inconsistent, and most cohorts were small with limited follow-up^10,11^. Our study aimed to evaluate cerebral, renal, and mesenteric oxygenation using NIRS in preterm neonates with hsPDA before and after treatment with ibuprofen or acetaminophen, with follow-up extended to 72 h.

## METHODS

### Study population

An observational prospective study was conducted in the third level Neonatal Intensive Care Unit (NICU) of Selçuk University Faculty of Medicine Hospital between September 2021 and November 2024. It was approved by the local ethics committee of Selçuk University Faculty of Medicine (2023/463). Informed consent was obtained from the parents before inclusion in the study. The diagnosis of PDA was made by transthoracic ECHO examination by two pediatric cardiologists after 72 h according to unit protocol. The study included premature neonates born between 24 and 34 weeks with a diagnosis of hsPDA. This was defined as a ductal diameter of >2 mm, a left atrial to aortic root ratio of ≥1.4, evidence of a left-to-right shunt, and decreased or absent diastolic flow in the superior mesenteric, middle cerebral, and/or renal arteries^11^. PDA patients not receiving medical treatment and underwent surgical ligation, premature (over 34 weeks) neonates, those with acute/chronic kidney disease, anomalies, genetic diseases, persistent pulmonary hypertension, or sepsis were excluded from the study. No formal power analysis was conducted. All subjects met the inclusion criteria and were consecutively enrolled.

### Drug administration protocol of neonates diagnosed with hemodynamically significant patent ductus arteriosus

Drug treatment was initiated following echocardiographic confirmation and clinical evaluation of a hsPDA in neonates. Eligible neonates were prospectively enrolled and then divided into two groups according to the drug administered: paracetamol (acetaminophen) or ibuprofen. Known contraindications to standard ibuprofen therapy for PDA closure were carefully evaluated prior to drug administration. In the absence of contraindications, patients received oral ibuprofen at a standard dose of 10 mg/kg for the first dose, followed by 5 mg/kg every 24 h for 3 days (total of three doses). Patients with contraindications to ibuprofen therapy received intravenous paracetamol (acetaminophen) at a dose of 15 mg/kg every 6 h for 7 days.

### Data collection

Clinical characteristics such as gestational age, birth weight, gender, multiple pregnancy, mode of delivery, prenatal steroid treatment, mechanical ventilation at the beginning of treatment, FiO_2_ during treatment, and the day PDA treatment was started were collected from all patients. In addition, ECHO findings [PDA diameter, Ratio of left atrium (LA)/aorta diameter, ejection fraction, fractional shortening, systolic and diastolic blood pressure (BP), and SpO_2_] values were recorded before and after treatment. A follow-up ECHO was obtained within 24 h of completing drug therapy.

According to the clinical protocol applied in the NICU, the diagnosis of hsPDA requiring treatment was established by ECHO, performed using a 7.5–12 MHz probe with the VIVID (GE Healthcare) and Philips EPIQ 7C (USA) echocardiography systems. All echocardiographic examinations were performed by two pediatric cardiologists in the supine position.

Arterial BP was measured noninvasively in the right arm using a PHILIPS MP40 neonatal BP monitor (Philips MP40, USA). All BP data were recorded 1 h before treatment (T0) and 24 h after the start of the first dose of drug infusion (T1), 24–48 h later (T2), and 48–72 h later (T3).

Regional tissue oxygenation measurements were performed in all patients during the treatment process using the NIRS device (INVOS 5100C Cerebral/Somatic Oximeter, Medtronic, USA). Neonatal/pediatric sensors (SenSmart 8004CB-NA non-adhesive probes with EQUANOX™ technology, Nonin Medical Inc., USA) were used. Each patient had a quadruple NIRS probe (cerebral, abdominal, right kidney, and left kidney) attached for the first 72 h of treatment. Data collection began 1 h before treatment and continued for 72 h during the treatment period. All NIRS data were recorded 1 h before treatment (T0) and 24 h after the start of the first dose of drug infusion (T1), 24–48 h later (T2) and 48–72 h later (T3). At the same time, SpO_2_ was measured to calculate FTOE, which reflects the balance between tissue oxygen delivery and tissue oxygen consumption and indirectly reflects tissue perfusion^12^. FTOE was calculated with the following formula: FTOE=(SpO_2_-rSO_2_)/SpO_2_.

### Statistical analysis

All analyses were performed using Statistical Package for the Social Sciences 23.0. Categorical data were summarized in frequency tables, and quantitative data in mean±standard deviation. The difference between the two groups in terms of numerical variables was investigated using Student's t-test and Mann-Whitney U-test. In repeated measurements of more than two groups, the repeated measures analysis of variance test was applied if the groups were normally distributed, and the Friedman test if they were not. Bonferroni correction was made in comparisons. Significance level was determined as p<0.05.

We performed an exploratory adjusted analysis using multivariable linear regression to account for potential confounding factors. Change in regional tissue oxygen saturation (ΔrSO_2_) from T0 (1 h before treatment) to T2 (24–48 h after treatment) was modelled as the dependent variable. The treatment group (ibuprofen versus paracetamol) was included as the main predictor, and gestational age, baseline mechanical ventilation, and baseline PDA diameter were included as covariates. The results are presented in Supplementary Table 1. Due to the small sample size, the number of covariates was limited to reduce overfitting.

## RESULTS

The study involved 24 patients with hsPDA who received drug treatment. Paracetamol and ibuprofen were used as monotherapies for closure therapy. Nine patients (37.5%) were given ibuprofen, and 15 patients (62.5%) were given paracetamol. The demographic and clinical characteristics of the patients included in the study are shown in Table 1. There were no significant differences between the groups in terms of these characteristics.

**Table 1:** Demographic and clinical characteristics of patients participating in the study.

		Paracetamol group n (%)	Ibuprofen group n (%)	Total n (%)	p
Gender n (%)	Male	5 (35.7)	9 (64.3)	14 (58.3)	0.831
	Female	4 (40.0)	6 (60.0)	10 (41.7)	
Mode of delivery (%)	NYVD	1 (20.0)	4 (80.0)	5 (20.8)	0.364
	C/S	8 (42.1)	11 (57.9)	19 (79.2)	
Antenatal steroid	Did not receive	4 (36.4)	7 (63.6)	11 (45.8)	0.916
	Received	5 (38.5)	8 (61.5)	13 (54.2)	
Oxygen type	Free O_2_	1 (50.0)	1 (50.0)	2 (8.3)	0.486
	BCPAP	2 (22.2)	7 (77.8)	9 (37.5)	
	Mechanical ventilation	6 (46.2)	7 (53.8)	13 (54.2)	
Red blood cell suspension	Not received	3 (33.3)	6 (66.7)	9 (37.5)	0.744
	Received	6 (40.0)	9 (60.0)	15 (62.5)	
Treatment outcome	Closure	3 (27.3)	8 (72.7)	11 (45.8)	0.614
	Narrowing	3 (42.8)	4 (57.2)	7 (29.2)	
	Insufficiency	3 (50.0)	3 (50.0)	6 (25.0)	
Total		9 (37.5)	15 (62.5)	24 (100.0)	
		Mean±SD	Mean±SD	Mean±SD	
Birth week		28.0±3.40	28.1±3.5	28.0±3.4	0.963
Weight		1,183.3±570.5	1,214.7±486	1,202.9±507.2	0.892
Mother's age		29.3±6.1	27.4±6.5	28.1±6.3	0.474

NYVD: normal vaginal delivery; C/S: cesarean section; SD: standard deviation; BCPAP: bubble continuous positive airway pressure.

The gestational age ranged from 23 to 34 weeks, with a mean of 28 weeks, and the mean birth weight was 1,202 g (min–max: 500–2,300 g). Maternal age ranged between 17 and 40 years.

Overall, after treatment with ibuprofen or paracetamol, DA was closed in 11 (45.8%) patients and narrowed in 7 (29.2%) patients. DA closure wasn't achieved in six patients (25.0%). The mean DA diameter was 3.24±0.7 mm before treatment and 1.63±1.5 mm after treatment. There was a statistically significant difference in DA diameter before and after treatment (p<0.001).

The mean PDA diameter of patients in the paracetamol group was 3.2 mm before treatment and 2.3 mm after, with no statistically significant difference (p=0.074). The mean PDA value of patients in the ibuprofen group was 3.2 mm before treatment and 1.6 mm after, with a statistically significant difference (p=0.002). Diastolic and mean BP values showed a statistically significant difference between the paracetamol and ibuprofen groups before and after treatment.

There was a statistically significant difference between the mesenteric rSO_2_ measurement values obtained at four different times (p=0.018). The mean value obtained at T0 was 60.33, while the mean values obtained at T1, T2, and T3 were 62.22, 63.43, and 63.32, respectively. The measurement value obtained at T0 was lower than the values obtained at other times. When we compared all patients according to different time intervals, there was no statistically significant difference in cerebral rSO_2_, cerebral FTOE, and SpO_2_, intestinal FTOE, right and left rSO_2,_ and FTOE values. The comparison of rSO_2_, FTOE, and SpO_2_ measurements at different time points is shown in Table 2.

**Table 2:** Comparison of regional tissue oxygenation, oxygen saturation, and fractional tissue oxygen extraction measurements at different time points.

	T0	T1	T2	T3	p
	Mean±SD	Mean±SD	Mean±SD	Mean±SD	
Systolic BP, mmHg	66.14±10.39	66.23±10.48	69.47±8.2	68.76±8.27	0.164
Diastolic BP, mmHg	37.73±9.76^a^	39.88±8.3^a^	42.5±8.76^a^	43.19±8.13^b^	**0.006**
Mean BP, mmHg	42.73±7.17^a^	46.95±5.71^b^	51.2±7.51^c^	51.17±8.23^c^	**<0.001**
Brain rSpO_2_	72.67±6.82	72.1±6.17	73.31±5.46	70.2±5.65	0.131
Mesenteric rSpO_2_	60.33±5.78^a^	62.22±5.95^b^	63.43±7.29^b^	63.32±7.05^b^	**0.018**
Right kidney rSpO_2_	69.58±10.18	68.2±7.81	67.99±7.15	68.18±8.15	0.728
Left kidney rSpO_2_	69.79±8.19	70.42±6.96	68.38±6.99	68.24±7.41	0.457
SpO_2_	94.58±2.58	94.48±1.86	93.74±1.64	93.92±1.99	0.162
Brain FTOE	0.23±0.07	0.24±0.08	0.22±0.06	0.25±0.06	0.201
Intestinal FTOE	0.36±0.09	0.34±0.06	0.32±0.08	0.33±0.07	0.104
Right kidney FTOE	0.26±0.10	0.28±0.08	0.27±0.07	0.27±0.08	0.613
Left kidney FTOE	0.25±0.08	0.26±0.07	0.27±0.07	0.27±0.08	0.358

rSpO_2_: regional tissue oxygenation, SpO_2_: oxygen saturation, FTOE: fractional tissue oxygen extraction; BP: blood pressure.

a–c No significant difference between time points with the same letter.

Bold values indicate statistical significance (p<0.05).

There was no statistically significant difference in rSO_2_, FTOE, and SpO_2_ values in tissues according to different time intervals in the paracetamol group (p>0.05). However, the mesenteric rSO_2_ values of the patients in the ibuprofen group were found to be significantly higher at T2 compared to T0 (p=0.01). Mesenteric FTOE values at T2 were lower than T0, but this difference was not statistically significant (p=0.700). See Table 3 for comparison of rSO_2_, SpO_2,_ and FTOE values according to different time intervals in paracetamol and ibuprofen group patients.

**Table 3:** Comparison of regional tissue oxygen saturation, oxygen saturation, and fractional tissue oxygen extraction values in patients in the paracetamol and ibuprofen groups at different time intervals.

Parameter	Paracetamol group					Ibuprofen group				p
	T0	T1	T2	T3	p	T0	T1	T2	T3	
Brain rSO_2_ (%)	73.22	72.72	73.77	70.13	0.200	72.33	71.72	73.02	70.23	0.340
Mesenteric rSO_2_ (%)	61.78	61.65	63.64	61.89	0.860	**59.47**	62.55	**63.29**	64.17	**<0.001**
Right kidney rSO_2_ (%)	68.56	68.1	68.16	65.17	0.26	70.2	68.25	67.88	69.98	0.450
Left kidney rSO_2_ (%)	71.3	68.74	68.6	66.8	0.33	68.87	71.42	68.24	69.11	0.058
SpO_2_ (%)	93.83	94.06	93.07	93.34	0.500	95.03	94.72	94.14	94.26	0.890
Brain FTOE (%)	0.21	0.22	0.2	0.24	0.530	0.23	0.24	0.22	0.25	0.450
Mesenteric FTOE (%)	0.34	0.34	0.31	0.33	0.70	0.37	0.33	0.32	0.31	0.120
Right kidney FTOE (%)	0.26	0.27	0.26	0	0.51	0.26	0.27	0.27	0.25	0.620
Left kidney FTOE (%)	0.24	0.26	0.26	0.28	0.32	0.27	0.24	0.27	0.26	0.056

rSpO_2_: regional tissue oxygenation; SpO_2_: oxygen saturation; rSO_2_: regional tissue oxygen saturation; FTOE: fractional tissue oxygen extraction.

Bold values indicate statistical significance (p<0.05).

## DISCUSSION

This prospective observational study assessed cerebral, renal, and mesenteric oxygenation by NIRS during pharmacological hsPDA closure. We observed a significant reduction in ductal diameter—more pronounced in the ibuprofen group—and a significant increase in mesenteric rSO_2_ during the 24–48-h interval after treatment initiation, while cerebral and renal oxygenation indices remained largely unchanged.

Navikien et al. reported significant decreases in ductal diameter after both paracetamol and ibuprofen treatment in hsPDA^11^. Consistently, we observed ductal narrowing following therapy, particularly with ibuprofen. However, meta-analyses suggest broadly comparable closure efficacy between paracetamol and ibuprofen, and differences across studies may reflect population characteristics, illness severity, and treatment selection^2^.

Our study found a statistically significant increase in BP after treatment in the ibuprofen group. Rios et al. state that hsPDA may be associated with circulatory disturbance and hypotension, and medical treatment may stabilize blood flow and pressure^13,14^. Some studies suggest that medical treatment in the presence of PDA can lead to higher BP^11,14^.

HsPDA is known to affect regional tissue oxygenation and circulation in the brain and kidneys, which are vital organs^5^. Our findings show that patients receiving ibuprofen had statistically significant increases in mesenteric rSO_2_ and decreases in mesenteric FTOE.

Some studies suggest that renal tissue oxygenation is affected earlier and more markedly by hsPDA than brain tissue^15,16^. This suggests that closure of the PDA primarily affects postductal circulation and oxygenation. It is known that shunt flow usually occurs in the direction affecting lower body circulation^17^. Brain tissue oxygenation is affected by preductal systemic perfusion, but renal oxygenation is more sensitive to diastolic leakage in the postductal region^18^. Our study found that shunt flow due to hsPDA affected the postductal region, with a statistically significant increase in mesenteric tissue oxygenation after medical treatment.

In contrast to our study, Poon et al. have reported an increase in cerebral rSO_2_ and a decrease in FTOE following PDA closure^19^. In some studies, only an increase in FTOE was found^12^. Navikienė et al. found a significant increase in renal rSO_2_ and a decrease in FTOE after hsPDA treatment, but didn't observe a significant change in cerebral tissue oxygenation^11^. Our study found a significant increase in mesenteric tissue oxygenation in neonates treated with ibuprofen for hsPDA, but no change in brain and renal tissue oxygenation. This may be due to cerebral autoregulation, which maintains constant blood flow despite fluctuations in BP^20^. Most cases of narrowed ductus have no significant changes to cerebral and renal rSO_2_ and FTOE. However, narrowing of the ductus has been shown to lead to favorable changes in mesenteric tissue oxygenation and circulation, which can be detected by NIRS. This suggests mesenteric tissue rSO_2_ may be a more sensitive indicator in assessing hsPDA treatment response compared with cerebral and renal rSpO_2_.

NIRS application times vary widely in the literature, with most studies lasting less than 1 h and only a few following for longer^16,19,21^. Our study's strength is its 72-h follow-up period, which contrasts with most studies’ shorter durations. Inconsistencies in the literature can largely be attributed to small sample sizes, which is an important limitation of our study too. Additionally, paracetamol was administered for up to 7 days, whereas NIRS monitoring covered the first 72 h. Consequently, our analysis primarily reflects early-phase physiological changes and may underestimate the effects of paracetamol over the full course. The study has several limitations, including a small sample size and non-randomized treatment allocation, since paracetamol was administered when ibuprofen was contraindicated. Therefore, residual confounding factors and limited statistical power—particularly with regard to cerebral and renal outcomes—may have contributed to the non-significant findings, which should be interpreted with caution.

## CONCLUSION

Ibuprofen treatment increased BP and improved oxygen in premature babies with hsPDA. Mesenteric tissue oxygenation may be a more sensitive indicator. More studies are needed.

## Data Availability

The datasets generated and/or analyzed during the current study are available from the corresponding author upon reasonable request.
