# Supplementary material for: Assessment of vital organ oxygenation by near-infrared spectroscopy in pharmacologic closure therapy for patent ductus arteriosus in preterm neonates
Source: Rev Assoc Med Bras (1992). 2026 Jun 29;72(4):e20251868. doi: 10.1590/1806-9282.20251868 (PMC13313075; doi:10.1590/1806-9282.20251868)
Supplement: Supplementary Table 1 [file 1806-9282-ramb-72-04-e20251868-suppl1.docx]

**Supplementary Table 1.** Adjusted analysis for change in regional tissue oxygenation from baseline (T0) to 24-48 hours (T2).

| **Outcome (ΔrSO_2_ T2–T0)** | **β (Ibuprofen vs. paracetamol)** | **95%CI (lower)** | **95%CI (upper)** | **p-value** | **Adj. R^2^** |
| --- | --- | --- | --- | --- | --- |
| Cerebral rSO_2_ change (T2–T0) | 1.10 | -3.65 | 5.86 | 0.633 | 0.040 |
| Mesenteric rSO_2_ change (T2–T0) | 1.63 | -3.90 | 7.15 | 0.545 | 0.234 |
| Right renal rSO_2_ change (T2–T0) | -4.17 | -13.90 | 5.57 | 0.382 | -0.100 |
| Left renal rSO_2_ change (T2–T0) | 0.83 | -7.69 | 9.35 | 0.841 | -0.058 |

rSO_2_: regional tissue oxygen saturation; PDA: patent ductus arteriosus; CI: confidence interval; T0, 1 hour before treatment; T2, 24–48 hours after treatment initiation. Model: Linear regression with ΔrSO_2_ (T2–T0) as dependent variable; predictors included treatment group (ibuprofen vs paracetamol), gestational age (weeks), mechanical ventilation at baseline (yes/no), and baseline PDA diameter (mm).
